# Alzheimer’s Disease-Related Cerebrospinal Fluid Biomarkers in Progressive Supranuclear Palsy

**DOI:** 10.3390/brainsci14090859

**Published:** 2024-08-26

**Authors:** Takanobu Ishiguro, Kensaku Kasuga

**Affiliations:** 1 Department of Neurology, Brain Research Institute, Niigata University, 1-757 Asahimachi-dori, Chuo-ku, Niigata 951-8585, Japan; 2 Department of Molecular Genetics, Brain Research Institute, Niigata University, 1-757 Asahimachi-dori, Chuo-ku, Niigata 951-8585, Japan; ken39@bri.niigata-u.ac.jp

**Keywords:** progressive supranuclear palsy, cerebrospinal fluid, β-amyloid, phosphorylated tau, total tau, neurofilament light chain

## Abstract

**Highlights:**

- Progressive Supranuclear Palsy (PSP) presents with various clinical phenotypes, making accurate diagnosis difficult.
- No biomarker systems, such as the AT(N) system for Alzheimer’s Disease (AD), have been established yet.
- In PSP, core AD cerebrospinal fluid biomarkers show a unique pattern, where Aβ42, Aβ40, p-tau, and t-tau levels are decreased while NfL levels are remarkably increased.

**Abstract:**

Progressive Supranuclear Palsy (PSP) is the most common four-repeat tauopathy. PSP cases are typically characterized by vertical gaze palsy and postural instability; however, various phenotypes have been reported, making antemortem diagnosis based on clinical symptoms challenging. The development of biomarkers reflecting brain pathology and the ability to diagnose patients based on these biomarkers are essential for developing future intervention strategies, including disease-modifying therapies. However, despite many dedicated efforts, no highly specific fluid biomarker for PSP has yet been established. Conversely, several cerebrospinal fluid (CSF) biomarkers of Alzheimer’s Disease (AD) have been established, and an AT(N) classification system has been proposed. Typically, among patients with AD, CSF amyloid β42 (Aβ42), but not Aβ40, is decreased, resulting in a reduction in the Aβ42/Aβ40 ratio, while tau phosphorylated at threonine 181 (p-tau181) and total tau (t-tau) are increased. Interestingly, the core CSF AD biomarkers show unique patterns in patients with PSP. Furthermore, reports have indicated that the CSF levels of both Aβ42 and Aβ40 are decreased independently of Aβ accumulation in PSP. Therefore, the Aβ42/Aβ40 ratio could potentially be used to differentiate PSP from AD. Additionally, studies have reported that CSF p-tau and t-tau are reduced in PSP, and that the neurofilament light chain is remarkably increased compared to healthy controls and patients with AD, even though PSP is a neurodegenerative disease associated with tau accumulation. These PSP-specific changes in AD-related core biomarkers may reflect the pathology of PSP and contribute to its diagnosis. As such, elucidating the mechanisms underlying the observed decreases in Aβ and tau levels could facilitate a better understanding of the pathogenesis of PSP.

## 1. Difficulties in the Antemortem Diagnosis of PSP

Progressive supranuclear palsy (PSP) is a primary tauopathy characterized by a four-repeat tau pathology [1]. Richardson’s syndrome (PSP-RS) [2], clinically characterized by vertical gaze palsy and postural instability, is a typical presentation, although various clinical phenotypes have been reported to date [1,3]. Of these, Progressive Supranuclear Palsy with predominant parkinsonism (PSP-P) presents with parkinsonism similar to Parkinson’s Disease (PD), showing levodopa responsiveness in its early stages; as such, its differentiation from PD crucial [4,5]. Further, patients with PSP-P have a longer survival prognosis than those with PSP-RS [6]. There are also various other phenotypes [1,3], such as PSP with predominant frontal presentation (PSP-F), characterized by frontal lobe symptoms similar to behavioral variant frontotemporal dementia [7,8]; PSP with predominant speech/language disorder (PSP-SL), which primarily involves speech and language disorders, including nonfluent/agrammatic primary progressive aphasia and progressive apraxia of speech [9,10]; PSP with predominant CBS (PSP-CBS), presenting with corticobasal syndrome [11,12]; PSP with progressive gait freezing (PSP-PGF), which predominantly features progressive gait freezing [13,14]; PSP with predominant ocular motor dysfunction (PSP-OM), marked by ocular motor dysfunction [6,15]; PSP with predominant with postural instability (PSP-PI), characterized by significant postural instability and frequent falls [6,16]; and PSP with predominant with cerebellar ataxia (PSP-C), characterized by a cerebellar ataxia phenotype, which highlights cerebellar symptoms, necessitating differentiation from multiple system atrophy (MSA) [17,18,19]. However, a significant proportion of patients progress towards the PSP-RS phenotype over time [1].

The first clinical diagnostic criteria for PSP were developed based on pathological diagnoses and published by the National Institute of Neurological Disorders and Stroke/Society for PSP (NINDS-SPSP) in 1996 [15]. These criteria can be used to detect PSP-RS with a high specificity due to the typical symptoms of vertical supranuclear gaze palsy and postural instability with falls in the first year. However, the sensitivity of this system was not high [15,20,21], as not all PSP cases present with the clinical features of PSP-RS in the early stages [1,6,21]. The Movement Disorder Society (MDS) criteria were proposed in 2017 [3]. These criteria aim to detect multiple clinical features of PSP early by evaluating four supporting clinical features (ocular motor dysfunction, postural instability, akinesia, and cognitive dysfunction), and assessing the PSP phenotype and its likelihood as suggestive, possible, or probable. The MDS criteria improved the sensitivity for PSP from 45.5% to 87.9% compared with the NINDS-PSPS criteria [20]. In cases where multiple phenotypes were fulfilled simultaneously, the multiple allocation extinction (MAX) rules were adopted [22]. If a patient presents with the PSP-RS phenotype, the pathology is likely PSP [6,15,23]. However, an autopsy-confirmed cohort reported that approximately 20% of cases diagnosed with probable or possible PSP using these clinical criteria did not have a PSP pathology [24]. Further, many autopsy-confirmed cases of PSP present with phenotypes other than PSP-RS, particularly in the early stages [1]. Of the antemortem phenotypes of autopsy-confirmed PSP cases, 24–54% were classified as PSP-RS, followed by 14–32% as PSP-P [4,6]. Thus, it is difficult to accurately detect the PSP pathology based solely on clinical symptoms. Clinical trials, such as those investigating anti-tau antibody treatments, have been initiated for PSP [25,26]. The early identification of a PSP pathology is important for effective therapeutic interventions, particularly as typical PSP-RS progresses faster than other types [27,28]. Differentiating PSP in the early stages from α-synucleinopathies, such as PD and MSA, is also essential for non-RS types.

Although the MDS criteria have improved the sensitivity of PSP diagnosis based on clinical symptoms, the accuracy of such diagnoses remains suboptimal for pathological detection [24,29], and achieving an accurate antemortem diagnosis remains a challenge. Furthermore, PSP commonly presents with a mixed pathology, with only approximately 8% of clinical cases presenting with a pure PSP pathology [30]. Therefore, the identification of biomarkers that enable the detection of early-stage pathologies is crucial for the development of disease-modifying therapies.

## 2. AT(N) Biomarkers in Alzheimer’s Disease

Alzheimer’s Disease (AD) is pathologically characterized by the extracellular deposition of β-amyloid (Aβ), as well as the intracellular accumulation of hyperphosphorylated tau [31]. Although both AD and PSP are tauopathies, in AD, Aβ pathology precedes the tau accumulation of isoforms with three microtubule-binding repeats and four repeats. As such, AD is classified as a secondary tauopathy.

The AT(N) classification system proposed by the National Institute on Aging and the Alzheimer’s Association is a useful and practical biomarker system for AD [32]. This AT(N) system employs either fluid or imaging biomarkers for Aβ deposition (A-marker), pathological tau (T-marker), and neurodegeneration/neuronal injury (N-marker) [32]. In this research framework, the cerebrospinal fluid (CSF) amyloid β 42 (Aβ42) and Aβ42/Aβ40 ratio is adopted as the A-marker, tau phosphorylated at threonine 181 (p-tau181) as the T-marker, and total tau (t-tau) as the N-marker. In AD, a specific pattern is observed, comprising a reduction in Aβ42 levels and the Aβ42/Aβ40 ratio, as well as elevations in p-tau and t-tau [32,33]. AD is defined in vivo when both the A and T markers are positive. These CSF biomarkers allow for highly accurate differentiation between groups with and without the AD pathology [34]. Recently, there have been several remarkable advances in novel CSF T-markers such as p-tau217 [35], N-markers such as neurofilament light chain (NfL) [36,37,38], and several blood biomarkers [39,40]. The AT(N) classification has since been revised to include markers of inflammation, vascular brain injury, and synucleinopathy in addition to the original AT(N) markers [41].

In contrast to the AT(N) biomarkers observed in AD, no disease-specific biomarkers enabling the early diagnosis of PSP have been established yet. However, some studies have analyzed the AT(N) biomarkers in clinically diagnosed non-AD neurodegenerative diseases, including PSP. These studies further revealed a unique pattern in the core CSF AD biomarkers, including Aβ42, p-tau181, and t-tau, in each neurodegenerative disease (Table 1). This section describes the mechanisms underlying the changes in AD-related biomarkers, and discusses how AD-related biomarkers are altered in patients with PSP.

## 3. Changes in Core AD biomarkers in PSP

### 3.1. Aβ: Aβ42, Aβ40

**Figure 1:**
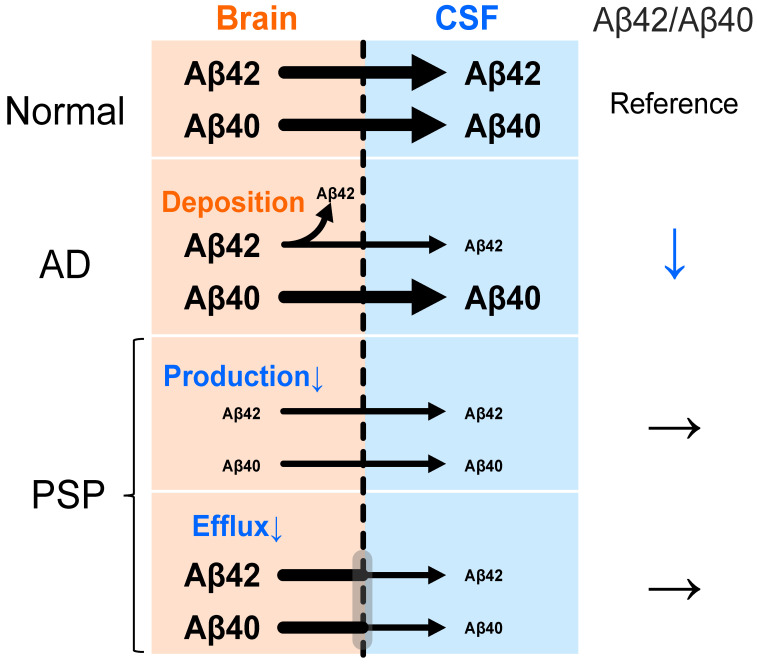
Differences in Aβ efflux and Aβ42/Aβ40 ratio in Alzheimer’s Disease and progressive supranuclear palsy. In Alzheimer’s Disease (AD), reflecting Aβ42 deposition as senile plaques, Aβ42 efflux from the brain to the cerebrospinal fluid (CSF) is reduced, but Aβ40 efflux is not changed. The Aβ42/Aβ40 ratio is decreased. In progressive supranuclear palsy (PSP), not only Aβ42, but also Aβ40, is reduced in the CSF, therefore, the Aβ42/Aβ40 ratio is not decreased. The speculated mechanisms are, regardless of Aβ species, that (1) Aβ production is reduced because of the decreased neural activity, (2) CSF dynamics are impaired and the Aβ efflux from the brain to the CSF is reduced.

In AD, the efflux of Aβ42 from the brain to the CSF is reduced [58], resulting in a decrease in CSF Aβ42 levels [32,33]. Aβ42 is a key component of senile plaques, while a reduction in its CSF levels has been associated with plaque density [59]. In contrast, Aβ40 levels have not been associated with the Aβ burden [60], resulting in a decrease in the Aβ42/Aβ40 ratio in AD, reflecting Aβ deposition (Figure 1). The CSF Aβ42/Aβ40 ratio is more effective at detecting the AD pathology than looking at Aβ42 alone, as the ratio can mitigate the effects of intra-individual variability and preanalytical factors [61].

It has further been reported that the CSF Aβ42 levels in clinically diagnosed PSP patients are either similar [42,43,48,50,51,52,54,55] or decreased [38,44,45,49,53] compared to healthy controls (Table 2). Notably, postmortem analyses revealed decreases in the levels of Aβ40 in addition to Aβ42 in PSP patients [60]. Kurihara et al. further reported that the CSF Aβ42 levels were decreased independently of the Aβ burden in autopsy-confirmed cases of PSP [62]. However, it should be noted that, even in cases primarily diagnosed with PSP, the Aβ42/Aβ40 ratio was reduced in cases with concurrent AD pathology [60]. Taken together, these results suggest that the Aβ42/Aβ40 ratio in PSP patients is comparable to that of controls, as both Aβ42 and Aβ40 are decreased independently of Aβ deposition (Figure 1), but that the Aβ42/Aβ40 ratio is decreased in patients with concomitant AD pathology.

Studies using both cell and animal models have shown that Aβ production is linked to neuronal activity [63]. Interestingly, cortical perfusion has been reported to be decreased in patients with PSP, in association with tau accumulation in the subcortical regions [64]. As such, the CSF Aβ levels may be reduced due to a decrease in neural activity associated with pathological tau accumulation in PSP. Alternatively, the CSF dynamics may be impaired in PSP, as prior studies have reported that CSF dynamic dysfunction, such as normal-pressure hydrocephalus, also reduces the Aβ levels in the CSF [65] (Figure 1). To clarify whether the decrease in Aβ in the CSF of PSP patients is caused by the decreased production of Aβ, decreased release of Aβ out of the cell, or another independent mechanism, it will be necessary to assess Aβ production and clearance using stable isotope labeling kinetics [58]. Recently, the concept of proteinopenia was proposed. Although the mechanism by which Aβ is decreased in PSP is not clear, it is possible that a decrease in the physiological protective effect of Aβ may influence PSP pathogenesis [66].

### 3.2. Tau: Total Tau (t-tau), Phosphorylated Tau (p-tau181)

**Figure 2:**
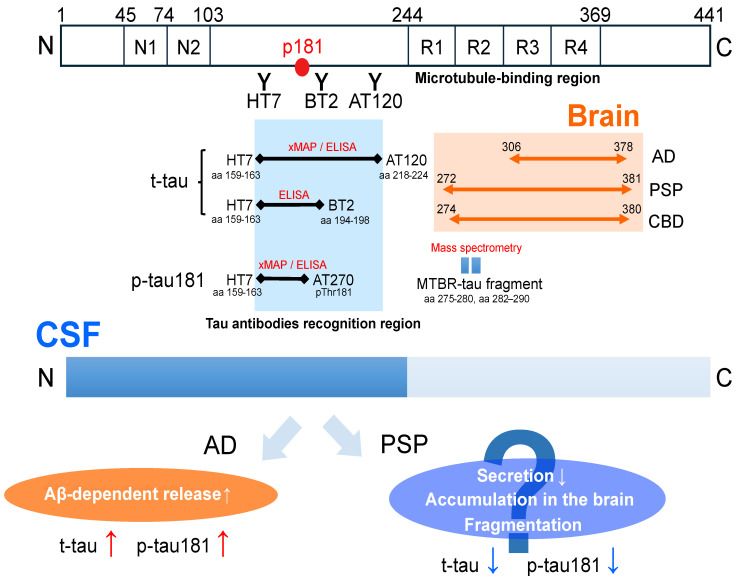
Tau protein detection and differentiation in Alzheimer’s Disease and progressive supranuclear palsy. Current platforms, such as xMAP and ELISA, combine several anti-tau antibodies, HT7, BT2, AT120, and AT270 (p181), to detect the middle region of tau in the cerebrospinal fluid (CSF). Pathophysiologically, the majority of extracellularly released tau are fragments extending from the N-terminus to the middle region, with truncated C-termini. In Alzheimer’s Disease (AD), progressive supranuclear palsy (PSP), and corticobasal degeneration (CBD), the core sequence of accumulated tau in the brain consists of the C-terminus of the microtubule-binding region (MTBR), which is not included in the N-terminal fragments of tau detected by t-tau and p-tau181 assays. In AD, the N-terminal fragment of tau in CSF increases depending on Aβ deposition, resulting in elevated t-tau and p-tau181 levels. In PSP, on the other hand, t-tau and p-tau181 levels are decreased. The speculated mechanisms underlying this are: (1) reduced tau secretion due to decreased neural activity, (2) accumulation of tau in the brain, and (3) progressive fragmentation of tau resulting in undetectable tau fragments. Mass spectrometry analysis has enabled the detection of MTBR-tau fragments (aa 275–280 and aa 282–290) in CSF, allowing for differentiation between PSP, CBD, AD, and frontotemporal lobar degeneration-*MAPT*.

In AD, the levels of both p-tau181 and t-tau are elevated [32,33]. Tau is predominantly expressed in neurons. Because t-tau in the CSF increases during acute neuronal damage, such as head trauma, cerebrovascular disease, and Creutzfeldt–Jakob disease, it is categorized as an ‘N-marker’ in the AT(N) system [32]. As such, elevated tau levels in the CSF were previously thought to reflect the extracellular release of tau following neuronal death. However, studies on cultured cells and mouse models have shown that tau, similar to Aβ, is physiologically released into the extracellular space in a neuronal-activity-dependent manner [67,68]. The majority of the tau detected in the human CSF comprises fragments extending from the N-terminus to the middle region, with the C-terminus being truncated [69,70] (Figure 2) and tau secretion increasing in response to Aβ deposition [69]. As such, the increased t-tau in the CSF in AD is thought to reflect an Aβ-dependent release rather than neuronal death. Full-length tau was not elevated in patients with AD compared to controls [70].

In AD, the elevation of CSF p-tau181 has been shown to be positively correlated with the spread of neurofibrillary changes [59], and is considered to be a characteristic change [34]. The phosphorylation site of p-tau181 is located in the middle region of tau, and mechanisms similar to an increase in t-tau have been suggested (Figure 2). In other words, p-tau181 detected in the CSF comprises fragments extending from the N-terminal to the middle region, and is considered to be increased, reflecting an Aβ-dependent release [69]. Studies using mouse models have further reported that Aβ deposition induces elevations in p-tau181 and t-tau in the CSF [71]. Notably, cryo-electronic microscopy experiments revealed that the core sequence of the neurofibrillary tangle was the C-terminus of the microtubule-binding region (MTBR) [72], which was not included in the N-terminal fragments detected as p-tau181 in the CSF (Figure 2). Therefore, CSF p-tau181 values are considered to reflect the Aβ-dependent hyperphosphorylation and fragmentation of tau, rather than tau accumulation itself.

In contrast, in PSP, the N-terminal fragment of tau in the CSF is decreased not only compared to that in AD patients, but also to that in healthy subjects [73]. Although one study indicated an increase [50], many studies have shown that the CSF t-tau and p-tau181 levels were either similar or reduced compared to those in controls in several cohorts based on clinical diagnosis [42,43,44,45,46,47,49,51,52,53,55,56,57,74] (Table 2). Additionally, although this has not been investigated in other PSP phenotypes, p-tau181 has been shown to be negatively correlated with the severity and progression of PSP-RS [75]. In an analysis of antemortem CSF biomarkers in autopsy-confirmed cases, the levels of both t-tau and p-tau were found to be decreased in PSP [60]. No such changes were observed in corticobasal degeneration (CBD), another primary four-repeat tauopathy [60]. As such, unlike in AD, the CSF tau levels are decreased in patients with PSP. The core sequence of tau accumulated in the brains with PSP and CBD, identified using cryo-electronic microscopy, partially overlaps with that in AD and is located at the C-terminus of the MTBR [76,77] (Figure 2). However, changes in the tau levels detected in the CSF of patients with PSP differ from those of patients with AD. Several hypotheses have been proposed to explain the reduction in the CSF tau levels: (1) Similar to speculation regarding the decrease in Aβ release/secretion, decreased neuronal activity may lead to a subsequent reduction in tau release/secretion, as tau has also been shown to be physiologically secreted extracellularly in a neural-activity-dependent manner [67,68]. (2) It may reflect tau accumulation in the brain. (3) Many tau fragments cannot be detected using current tau antibody platforms. Thus, enhanced tau cleavage due to increased appoptosin and caspase-3-dependent activation may generate undetectable tau fragments [75,78]. Given these multiple theories, there is significant interest in elucidating the distinct molecular pathogenesis of PSP, which differs from that of AD. Recently, a mass spectrometry analysis of CSF four-repeat isoform-specific tau fragments in MTBR showed promise in distinguishing PSP from CBD, AD, and frontotemporal lobar degeneration-*MAPT* [79] (Figure 2). Seed activity amplification, which specifically amplifies pathologically aggregated seeds, has also been reported to be useful in the diagnosis of PSP [80]. Disease-specific tau accumulation markers are also expected to provide new insights into this field. Furthermore, studies using tau PET have revealed accumulation in the globus pallidus, subthalamic nucleus, and dentate nucleus of the cerebellum in PSP patients [81,82,83]. Although a moderate correlation between cortical tau accumulation and p-tau levels in AD has been reported [84], its correlation with PSP remains unclear. As such, confirming the association between CSF p-tau and tau PET accumulation may be helpful in clarifying whether the reduction in the CSF p-tau181 in PSP reflects tau accumulation.

### 3.3. NfL

CSF NfL is a valuable biomarker of neurodegeneration and neuronal injury [36,37,38]. Both t-tau and NfL have been used as N markers. However, they reflect different aspects of neurodegeneration. As mentioned above, t-tau detects the N-terminal fragments of tau released extracellularly in association with the Aβ pathology, whereas NfL nonspecifically reflects neurodegeneration and neuronal damage [36]. Although changes in the t-tau and p-tau181 levels have been shown to be highly correlated, changes in the t-tau and NfL levels have not [85,86]. In PSP, the NfL levels are strongly correlated with disease severity and progression, making it a useful assessment tool [75]. However, NfL is nonspecifically elevated in many neurological disorders [36], making the diagnosis of PSP using this marker alone difficult. In PSP, unlike the t-tau levels, the NfL levels were significantly increased in several cohorts compared to those in healthy controls [42,43,44]. Therefore, an ‘N-marker mismatch’ between t-tau and NfL exists in PSP, which could be a distinguishing biomarker pattern of the disease. NfL is expressed in large-caliber myelinated fibers [37], suggesting that PSP may cause a more severe impairment of myelinated fibers than AD. A recent tau positron emission tomography study showed that tau accumulation was lower in highly myelinated cortical regions in AD [87]. Therefore, an analysis of the association between myelination and tau-accumulated regions in PSP may reveal the cause of this N-marker mismatch. Future studies should clarify whether N-marker mismatches are specific to PSP.

## 4. Conclusions

In this article, we reviewed the distinct changes in core AD CSF biomarkers observed in PSP, in which both Aβ and tau molecular species showed reductions, but NfL was extremely elevated. These findings could potentially serve as discriminative markers distinguishing PSP from other atypical Parkinsonian syndromes, which could aid in its diagnosis. The physiological mechanisms associated with Aβ and tau production/secretion/clearance in the brain indicate a potential impact of neuronal activity on the changes in CSF biomarkers in PSP, providing insights into the pathological mechanisms of this disease. These findings provide crucial clues regarding the pathophysiology of PSP.

It is important that future research be conducted to discover highly specific biomarkers for PSP. In addition, it may be equally important to combine changes in multiple biomarkers to allow for a comprehensive understanding of this condition to improve its diagnostic accuracy. A singular change in Aβ42, Aβ40, t-tau, p-tau181, or NfL is insufficient for the diagnosis of AD alone. The same applies in patients with PSP. Although each biomarker has limitations in diagnostic precision, understanding individual changes and combining multiple biomarkers could significantly enhance diagnostic accuracy. For example, one recent study showed that combining serum NfL levels with MRI findings of third ventricle enlargement could be used to differentiate PSP from PD and healthy controls with a high accuracy [88]. As with the AT(N) system in AD biomarkers [32], understanding PSP pathology through a combination of multiple biomarkers is crucial. Therefore, this approach highlights the importance of using multiple biomarkers to improve diagnostic precision and comprehend specific changes in individual biomarkers. The development of disease-modifying therapies for AD can be attributed to the success of early diagnosis by using biomarkers [89,90]. Similarly, in PSP, the use of biomarkers to facilitate more accurate early diagnosis is expected to contribute to the success of clinical trials [91]. Therefore, in addition to identifying PSP-specific biomarkers, a comprehensive approach for evaluating multiple biomarkers is essential for the development of future disease-modifying therapies.

## Figures and Tables

**Table 1:** Cerebrospinal fluid biomarker studies in neurogenerative diseases based on clinical diagnosis.

Study [Reference]	Platform	Cohort Sizes (n)			
		CTRL	AD	CBS/CBD	PSP
Magdalinou NK et al. [42]	ELISA	30	26	14	33
Hall S et al. [43]	xMAP	107	48	12	45
Hansson O et al. (original) [44]	ELISA	53	n/a	5	19
Hansson O et al. (validation) [44]	ELISA	26	n/a	12	29
Wagshal D et al. (original) [45]	xMAP	26	37	n/a	24
Thijssen EH et al. [46]	xMAP	69	56	39	48
Urakami K et al. [47]	ELISA	36	n/a	27	30
Holmberg B et al. [48]	ELISA	32	n/a	n/a	15
Noguchi M et al. [49]	ELISA	43	69	9	18
Aerts MB et al. [50]	ELISA	49	n/a	12	21
Schoonenboom NS et al. [51]	ELISA	275	512	16	20
Scherling CS et al. [52]	xMAP	47	50	17	22
Bäckström DC et al. [53]	ELISA	30	n/a	n/a	12
Constantinides VC et al. [54]	ELISA	18	n/a	17	19
Jeppsson A et al. [55]	Aβ42: ECL	54	50	15	34
	t-tau, p-tau181: ELISA				
Borroni B et al. [56]	ELISA	27	29	20	21
Snellman A et al. [57]	ELISA	22	22	n/a	22

Abbreviations: ELISA, Enzyme-Linked Immunosorbent Assay; xMAP, Multiplexed Assay Platform; ECL, Electrochemiluminescence; Aβ42, β-amyloid42; t-tau, total tau; p-tau181, phosphorylated tau at threonine 181; CTRL, healthy controls; PSP, progressive supranuclear palsy; AD, Alzheimer’s disease; CBS, corticobasal syndrome; CBD, corticobasal degeneration; n/a, data not available.

**Table 2:** Significant changes in cerebrospinal fluid Aβ42, t-tau, and p-tau181 for degenerative disease patients compared to normal controls.

Study [Reference]	AD			CBS/CBD			PSP		
	Aβ42	t-tau	p-tau181	Aβ42	t-tau	p-tau181	Aβ42	t-tau	p-tau181
Magdalinou NK et al. [42]	↓ *	↑ *	↑ *	↔	↔	↔	↔	↔	↔
Hall S et al. [43]	↓ ^‡^	↑ ^‡^	↑ ^‡^	↔	↔	↔	↔	↔	↓ ^‡^
Hansson O et al. (original) [44]	n/a	n/a	n/a	↔	↔	↔	↓ *	↓ *	↓ ^†^
Hansson O et al. (validation) [44]	n/a	n/a	n/a	↔	↔	↔	↓ *	↔	↓ ^†^
Wagshal D et al. (original) [45]	↓ *	↑ *	↑ *	n/a	n/a	n/a	↓ *	↓ *	↓ *
Thijssen EH et al. [46]	n/a	n/a	↑ *	n/a	n/a	↔	n/a	n/a	↔
Urakami K et al. [47]	n/a	n/a	n/a	n/a	↑ ^‡^	n/a	n/a	↔	n/a
Holmberg B et al. [48]	n/a	n/a	n/a	n/a	n/a	n/a	↔	n/a	n/a
Noguchi M et al. [49]	↓ ^‡^	↑ ^‡^	↑ ^‡^	↓ *	↔	↔	↓ ^‡^	↔	↔
Aerts MB et al. [50]	n/a	n/a	n/a	↔	↑ ^‡^	↑ ^†^	↔	↑ *	↔
Schoonenboom NS et al. [51]	↓ *	↑ *	↑ *	↔	↔	↔	↔	↔	↔
Scherling CS et al. [52]	↓^‡^	↑ *	↑^‡^	↔	↔	↔	↔	↔	↔
Bäckström DC et al. [53]	n/a	n/a	n/a	n/a	n/a	n/a	↓ *	↔	↔
Constantinides VC et al. [54]	n/a	n/a	n/a	↔	↑ *	↔	↔	↔	↔
Jeppsson A et al. [55]	↓ ^‡^	↑ ^‡^	↑ ^‡^	↔	↔	↔	↔	↔	↔
Borroni B et al. [56]	n/a	↑ ^‡^	↑ ^†^	n/a	↔	↔	n/a	↔	↔
Snellman A et al. [57]	↓^‡^	↑ ^‡^	↑ ^‡^	n/a	n/a	n/a	↔	↔	↔

Compared to healthy controls, ‘significant increase ↑’, ‘significant decrease ↓’, ‘there is no significant difference ↔’. * *p* < 0.05, ^†^ *p* < 0.01, ^‡^ *p* < 0.001. Aβ42, β-amyloid42; t-tau, total tau; p-tau181, phosphorylated tau at threonine 181; PSP, progressive supranuclear palsy; AD, Alzheimer’s disease; CBS, corticobasal syndrome; CBD, corticobasal degeneration; n/a, data not available.

## Data Availability

No new data were created or analyzed in the preparation of this review.
